# An all-Africa dataset of energy model “supply regions” for solar photovoltaic and wind power

**DOI:** 10.1038/s41597-022-01786-5

**Published:** 2022-10-31

**Authors:** Sebastian Sterl, Bilal Hussain, Asami Miketa, Yunshu Li, Bruno Merven, Mohammed Bassam Ben Ticha, Mohamed A. Eltahir Elabbas, Wim Thiery, Daniel Russo

**Affiliations:** 1 International Renewable Energy Agency (IRENA), Bonn, Germany; 2 Faculty of Engineering, BClimate group, Department HYDR, Vrije Universiteit Brussel, Brussels, Belgium; 3 World Resources Institute (WRI), Regional Hub for Africa, Addis Ababa, Ethiopia; 4 Energy Systems Research Group, University of Cape Town, Cape Town, South Africa; 5 International Atomic Energy Agency (IAEA), Vienna, Austria; 6 Institute for Research in Technology (IIT), ICAI School of Engineering, Comillas Pontifical University, Madrid, Spain

**Keywords:** Energy modelling, Solar cells, Wind energy, Power stations, Energy grids and networks

## Abstract

With solar and wind power generation reaching unprecedented growth rates globally, much research effort has recently gone into a comprehensive mapping of the worldwide potential of these variable renewable electricity (VRE) sources. From a perspective of energy systems analysis, the locations with the strongest resources may not necessarily be the best candidates for investment in new power plants, since the distance from existing grid and road infrastructures and the temporal variability of power generation also matter. To inform energy planning and policymaking, cost-optimisation models for energy systems must be fed with adequate data on potential sites for VRE plants, including costs reflective of resource strength, grid expansion needs and full hourly generation profiles. Such data, tailored to energy system models, has been lacking up to now. In this study, we present a new open-source and open-access all-Africa dataset of “supply regions” for solar photovoltaic and onshore wind power to feed energy models and inform capacity expansion planning.

## Introduction

Globally, the deployment of modern renewable electricity sources has reached unprecedented levels, mainly driven by a strong growth of solar photovoltaic (PV) and wind power generation^1^. The typical levelised cost of electricity (LCOE) of solar PV and wind power projects has dropped substantially and resulted in cost-competitiveness with fossil fuel and hydropower plants^2^. Among other drivers, this has paved the way for high penetrations of solar PV and wind power in various countries’ electricity mixes, such as Denmark, Germany and Uruguay^3^. As a consequence of declining costs—a trend that is projected to continue—long-term capacity expansion planning at a national and regional level, based on cost-optimisation procedures, often suggests solar PV and wind power as priorities for future capacity buildout^4^.

Solar PV and wind power have a specific characteristic in which they differ from more traditional methods of power generation: their electricity yield varies in function of meteorological parameters such as irradiation, temperature and wind speed. Solar PV and wind power, classified as variable renewable electricity (VRE) resources, exhibit weather-related variability on all timescales from sub-hourly to interannual, and their yield is site-specific. A power system with a high share of VRE necessitates increased power system flexibility to cope with these variabilities^5^. Long-term energy planning, and models used therein, therefore need to take these aspects into consideration^6^. When planning future power systems with potentially high shares of VRE using such models, it is therefore particularly important to properly represent site-specific characteristics of VRE, including site-specific hourly, seasonal and interannual variabilities, and site-specific costs accounting for additional grid and road infrastructure needs as a function of the distance between each site and existing infrastructure^7^.

In order to represent VRE investment options whose characteristics differ across space, capacity expansion models theoretically require a comprehensive set of potential VRE plant sites as input to allow well-informed planning, each with their own temporal generation profile, similar to representing site-specific hydropower options in these models^8^. While datasets such as the Global Solar Atlas and the Global Wind Atlas^9,10^ provide a comprehensive mapping of VRE potential across the world at high resolution, they do not provide the associated infrastructure costs of power plant deployment for each site, and neither would it be practical to feed cost-optimisation models with all sites (pixels) in a given region without any prior cost-based screening, for reasons related to computational performance. At the same time, using lighter datasets based e.g. on existing projects as a proxy for an entire region is also of limited use, as model results would not provide any information on preferences for VRE deployment across different potential sites. For instance, such an approach would not elucidate the effect of site distance from grid infrastructure on costs^11^. Clearly, a representative subset of attractive sites for VRE deployment would be the preferred option to feed into capacity expansion models.

Each site in such a subset would have to be attributed to its own resource strength, temporal variability and associated grid and road network expansion costs. Once fed into a capacity expansion model, this would allow elucidating the optimal deployment of VRE plants, i.e. a portfolio of solar and wind power plants across the most appropriate locations. For the African continent, whose burgeoning power systems imply a chance to plan power grids from the outset to accommodate VRE^5^, the need for such modelling exercises is especially high.

This need is accentuated by the fast-growing deployment of VRE plants in different parts of the African continent, and the projected continuation of this deployment over the next decade. Currently, the deployment of solar PV and wind power in Africa is roughly evenly matched, with installed capacities of solar PV at around 8 GW as of 2020–21^12^, and wind power at 6.5 GW^13^. For solar power, this number is strongly dominated by South Africa and Egypt, which cover around 80% of installed capacity on the continent^12^. For wind power, the capacities are somewhat more spread out: South Africa, Egypt and Morocco record nearly two-thirds, with the remaining one-third mostly in Tunisia, Kenya, Ethiopia and Mauritania^13^. Given the favourable cost projections for both solar PV and wind power, the International Energy Agency predicts that these sources could record strongly increased growth rates across Africa in the period up to 2030, and reach 27% of Africa’s aggregate electricity mix by that same year^14^.

One attempt to provide such subsets of attractive VRE plant sites, focused on the Eastern and Southern African regions, was previously published in literature^15,16^. However, this methodology focused exclusively on near-grid resources (within 50–100 km of existing grid infrastructure) and did not calculate annual yields of VRE in a bottom-up manner based on open-source hourly meteorological conditions.

This study presents an attempt to go beyond refs. ^15,16^ by developing and open-sourcing a full workflow of creating spatiotemporally model-ready VRE investment options for Africa based on publicly available data. We present a novel representative subset of attractive sites for solar PV and onshore wind power for the entire African continent. Hereafter, we refer to these sites as “Model Supply Regions” (MSRs). This MSR dataset was created from an in-depth analysis of various existing datasets on resource potential, grid infrastructure, land use, topography and others (see Methods section “Additional methodological details”), and achieves hourly temporal resolution and kilometre-scale spatial resolution. This dataset fills an important research need by closing the gap between comprehensive datasets on African VRE potential (such as the Global Solar Atlas and Global Wind Atlas) on the one hand, and the input needed to run cost-optimisation models on the other. It also allows a detailed analysis of the trade-offs involved in exploiting excellent, but far-from-grid resources as compared to mediocre but more accessible resources, which is a crucial component of power systems planning to be elaborated for many African countries. The rest of the paper is organised as follows: we describe the overall approach in its main steps, with more details provided in the Methods section “Additional methodological details”. This is followed by a section that presents results obtained, before concluding with a discussion, conclusions and future work section.

## Methods

### Modelling flowchart

**Fig. 1:**
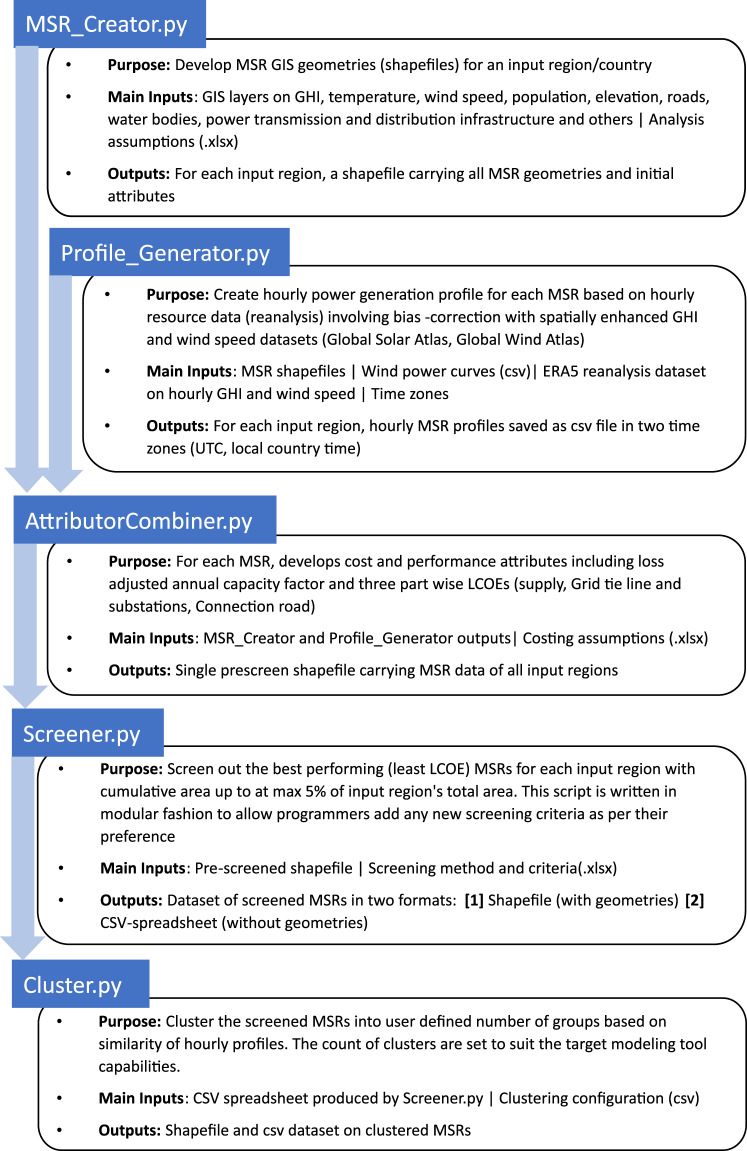
The MSR toolset comprises of five Python scripts that are run sequentially. A high-level description of each script and process flow is illustrated in this figure.

The principle of MSR creation is based on the combination of various geospatial datasets to lead to a representative subset of sites that can, in practical terms, be considered attractive sites for VRE plant deployment. A flowchart of the modelling process described in this section is given in Fig. 1. The model is implemented five Python-based scripts which execute the different stages described below: MSR creation, hourly profile generation, attribution, screening and clustering.

### Creating model supply regions (MSRs)

**Fig. 2:**
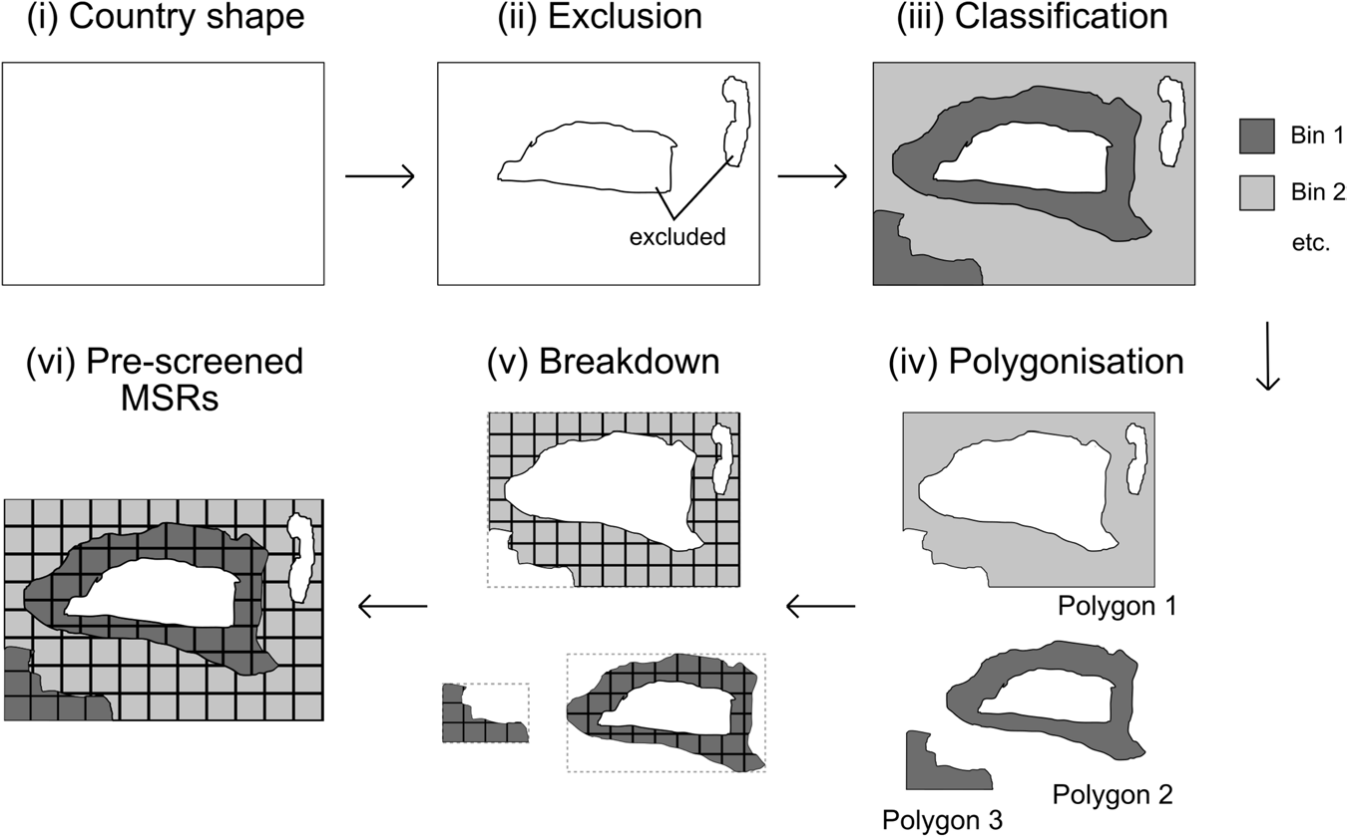
Process of MSR creation. This schematic shows the various steps of MSR creation (see also Methods), starting from (i) the boundaries of a hypothetical rectangular country, through (ii) the exclusion of unsuitable areas, (iii) the classification of the suitable areas into different bins representing VRE resources of different strength, (iv) the polygonization of the areas in each bin, and (v) the breakdown of each polygon into smaller cells, to arrive at (vi) a collection of pre-screened MSRs, each with their own specific characteristics, for the country.

The process of MSR creation is indicated schematically in Fig. 2 for a hypothetical rectangular country (panel (i) in Fig. 2), and summarised hereafter. Starting from the map of the African continent, the following parameters are used to select a geographically referenced subset of sites within each country (details are given in the Methods section “Additional methodological details”):

#### Resource strength

Only sites where average VRE resources (irradiation and wind speed) are above a certain minimum threshold, typical for commercial exploitation, are considered for inclusion.

#### Population density

Very densely populated areas, e.g. cities, are excluded from consideration.

#### Elevation

Locations above a certain elevation are excluded.

#### Slope

Locations with a slope beyond a given threshold are excluded.

#### Land use

Sites are only considered if they fall within certain categories of land use.

#### Protected areas

Natural reserves and other protected sites are excluded.

#### Distance from roads

Only sites within a certain vicinity of existing road networks are considered.

The above criteria define the exclusion areas, and only regions that meet all criteria are considered in the subsequent steps as potential areas for VRE deployment (ii).

These inclusion areas contain geographically close areas with steep resource gradients. To separate areas of different resource strength from each other, each country’s map of inclusion areas is first classified into five bins (iii) that reflect VRE resources of different strength (see Methods section “Additional methodological details”). The binned areas are then polygonised (iv), i.e. marking boundaries around contiguous included sites, to define a set of contiguous areas that belong to the same bin.

The data in each bin is then separately broken down (v) by applying a raster consisting of equally sized square cells onto each bin’s polygon features (see Methods section “Additional methodological details”). The raster cell area is based on a pre-defined maximum capacity threshold (in GW) of the technology in question, chosen to correspond to typical high voltage transmission level power evacuation infrastructure, and is calculated using the typical spatial footprint (MW/km^2^, see Methods section “Additional methodological details”) of solar PV or wind power plants. This breaks the polygon features in each bin down into smaller cells with a clearly defined maximum size (see Methods section “Additional methodological details”).

### Attribution

Each of these cells is defined as an individual MSR (vi) with its own specific attributed parameters such as maximum deployable capacity (in MW) based on cell area, average capacity factor (CF), distance from grid and road infrastructure, resource strength, hourly VRE generation profile, etc. For a complete list of attributes, the reader is referred to Supplementary Material A. The methodology to obtain hourly VRE generation profiles is described in detail in section “Additional methodological details” below.

This approach yielded a total of 79,608 MSRs for solar PV and 36,352 MSRs for wind, corresponding to 56 TW and 29 TW of generation capacity, respectively. In theory, MSRs can now be directly input as individual investment options in capacity expansion models, similar to how hydropower can be represented with site-specific investment options. However, depending on the scope of the modelling and the computational power available, the numbers of the MSR may be unpractical for direct use.

Therefore, we developed two further steps aiming at reducing the computational requirements by (1) pre-selecting the most desirable MSRs for model inclusion (“screening”) and (2) grouping MSRs with similar characteristics together as a single investment option (“clustering”). These are described next.

### The screening and clustering of MSRs for energy modelling applications

In this study, the screening step was based on the expected LCOE of each MSR. This LCOE was defined to include not only the costs of potential power plant construction and operation & maintenance in the MSR, but also additional costs for substation and transmission line and road construction costs for grid connection of the MSR (see Supplementary Material B). The MSRs were then ranked from lowest to highest expected LCOE. This allowed to screen out a high-ranking sub-selection of the MSRs in each country based on LCOE, such that this subset (the “best” sites in LCOE terms) can be used in capacity expansion planning.

In this analysis, we screened the dataset according to the criterion that the total area of screened MSRs should not exceed 5% of an individual country’s surface area. Since screening criteria can be arbitrarily defined, we note that this 5% is purely meant for demonstration purposes. Other criteria (e.g. “the cheapest 45 MSRs per country”, “all MSRs, ranked by LCOE, whose annual power generation would be equal to the country’s electricity demand”, etc.) could be equally or more valid, depending on the research question or the policy objective. Overall, the data set is designed such that a range of criteria for selecting an optimal MSR subset can be easily implemented.

The MSRs screened for this analysis are provided in Excel files along with their metadata, which includes full hourly profiles and assumed costs, and also as shapefiles (see Data Availability). These are intended to serve the energy modelling community to provide a comprehensive set of potential VRE plants, representing the lowest-LCOE sites within each country, as inputs to cost-optimisation models. Since all MSRs come with their own specific costs and own temporal availability profiles, they can be distinguished as separate technologies in such models (and their cost parameters potentially further refined). The analysis presented in the remainder of this paper is based on this screened dataset. Users wishing to use alternative screening criteria are invited to use the open-sourced Model Supply Regions code (see Code Availability) and adapt it to their needs.

Since screening criteria can be arbitrarily defined, the number of MSRs provided by the screening may still be too high for computational purposes (in this case of screening up to 5% of a country’s area, the number of MSRs is still several thousand). In particular, many neighbouring MSRs may have very similar temporal profiles and very similar costs, potentially leading to long runtimes of optimisation models. To mitigate this issue, one could simply limit the screening further. However, the latter could lead to a loss of information on the different types of resource profiles present within a country.

Therefore, a further clustering method is proposed based on the mathematical technique of *k-*means clustering, which allows to numerically group MSRs with extremely similar profiles within a country into a user-defined number of “clusters”, summing up their overall potential and calculating aggregated temporal profiles and costs. While this arguably leads to the loss of some spatial granularity, it preserves a wide range of profiles for inclusion in energy models while allowing practically feasible runtimes. The approach is explained in more detail in the Methods section “Additional methodological details” (cf. also Supplementary Material E), and a corresponding clustering script is included in the open-sourced Model Supply Regions code.

While our screening method is based on costs (LCOE), “strongest” and “cheapest” resources are not synonymous (cf. Figure 4c,d below). In addition, we note that “cheapest” and “optimal” are not synonymous either. Cost-optimisation models would not automatically prefer the very cheapest MSRs when selecting from either VRE technology. Notably, the temporal characteristics of power generation potential, especially diurnal and seasonal profiles, can differ strongly between MSRs. Since cost-optimisation is done on the basis of the entire system under study^11^, it is likely that cases exist where it is preferable to deploy VRE plants in locations where resources are *neither the strongest nor the cheapest*—e.g. in locations where the seasonality of the resource has the best fit with other elements of the system (such as complementarity with other resources, with demand, or with storage needs). For these reasons, we note that a static comparison of MSRs’ LCOE only serves for screening, and does not replace the need for further analysis of “optimality” using capacity expansion models.

The following section provides deeper methodological details on the above-described steps of MSR creation. It is followed by a results section which focuses on an analysis of the screened MSRs that can be used as input to capacity expansion models. It does not include an analysis of the application of the MSR in such models themselves.

### Additional methodological details

#### Datasets used in MSR creation

Average resource strength data for solar PV was obtained from the Global Solar Atlas^9^ at 1 × 1 km^2^ resolution; data for wind power was obtained from the Global Wind Atlas^10^ at 250 × 250 m^2^ resolution. The inclusion threshold (lower limit) for solar PV resources was an annual average Global Horizontal Irradiation (GHI) of 4 kWh/m^2^/day; the inclusion threshold for wind resources was an annual average wind speed of 6 m/s at 100 m height.

Population density was obtained from the Oak Ridge National Laboratory’s LandScan 2019 dataset^17^ at 1 × 1 km^2^ resolution, with an exclusion threshold (upper limit) of 100 inhabitants/km^2^.

Elevation was obtained from the Shuttle Radar Topography Mission (STRM) at 30 × 30 m^2^ resolution^18^, with an exclusion threshold (upper limit) of 2000 m above sea level. Slopes were obtained from the same dataset (Slope = [difference in elevation between two points]/[Distance between two points] * 100%), with an exclusion threshold of 20%.

Land use maps were obtained from the European Space Agency’s GlobCover (2009) map^19^ at 300 × 300 m^2^. The included land cover categories are 11, 14, 20, 30, 110, 120, 130, 140, 150, 180, 190, and 200.

Protected areas were obtained from the World Database on Protected Areas^20^.

Existing transmission grid infrastructure was obtained from the GridFinder (2020) dataset^21^ and the existing road network from the Global Roads Inventory Project (GRIP)^22^. No exclusion threshold was considered for the distance of a site to existing grid infrastructure. An upper limit of 50 km was used for the distance between a site and the road network.

The search for nearest transmission and road infrastructure was done considering exclusively the infrastructure within a country’s borders. Only in cases where a country did not have one of such infrastructure types within its borders, the search for that infrastructure type was relaxed to its neighbouring countries.

The GRIP dataset provides roads in different categories, e.g. “primary”, “secondary”, etc. (to distinguish e.g. highways from dirt roads). The MSR code provides the flexibility to select any subset of these categories. In the present paper, all GRIP road categories were included in the search.

The different resource bins for MSR creation were obtained by dividing the range between the lower limit used for the resources and the maximum resource value observed across non-excluded areas into five equally spaced bins. The resource bins are thus country-specific.

The breakdown of the polygons in each resource bin happens by bounding each polygon with a rectangle, and subsequently dividing this rectangle vertically and horizontally into equidistant rows and columns. The number of rows and columns is determined by dividing the vertical and horizontal dimensions of the rectangle by the side length of a square representing the maximum deployable capacity in each MSR (see below), and rounding this number down to the nearest integer. Each unit of the polygon enclosed by vertical and horizontal lines and, in certain cases, the polygon boundary, is defined as an MSR.

The maximum deployable capacity in each MSR (i.e. the typical size of a VRE power plant, which defines the maximum MSR area) was taken to be 2.7 GW (based on evacuation with a four-circuit 500 kV line, with a single 500 kV line assumed capable of evacuating up to 900 MW and one extra line included for N-1 security^15^). The spatial footprint of solar PV and wind power plants was taken to be 33 MW/km^2^ for solar PV^23,24^ and 11.8 MW/km^2^ for wind power^25^. In addition, we assumed that investing parties would not allocate all the area within an MSR to solar panels or wind turbines (given the need for various types of infrastructure, such as on-site roads), and used an area discount factor (the percentage of an MSR actually used for the power plant itself) of 10% for solar PV and 25% for wind^25^. The maximum area of an MSR was thus 818 km^2^ for solar PV, and 915 km^2^ for wind.

Very small MSRs that would represent a capacity potential (MW) below the user-specified minimum size of (in this case) 20 MW were discarded. This criterion is set to ensure that all MSRs represent a substantial enough utility-scale power evacuation opportunity.

A list of cost parameters (installation, operation and maintenance of power plants, transmission lines, substations and road extension) used in the calculation of LCOEs is provided in Supplementary Material B.

We note that the code for all steps of MSR creation has been made publicly available along with this paper (see Code Availability), and hence, could be readily re-run with other parameter values should future users wish to do so.

#### Temporal profiles of VRE generation

While average resource strength can be inferred at high spatial resolution from the Global Solar Atlas and Global Wind Atlas, these databases do not provide hourly data series of potential power generation or capacity factors. We obtained hourly data series of Global Horizontal Irradiation (GHI), ambient temperature, and 100-m wind speed from the ERA5 reanalysis dataset^26^, whose spatial resolution is coarser at 31 × 31 km^2^. For each MSR, the hourly profiles of these parameters were extracted from the nearest ERA5 cell through Nearest Neighbour spatial interpolation, based on centre-to-centre-distance between the MSR in question and the ERA5 cell. We used the meteorological year 2018 to perform the calculations as an example, noting that any other year or an average across multiple years could be used as well. After extracting these data, the GHI and wind speed datasets (8760 values, representing all hours in a year) were bias-corrected to the respective annual average values across MSRs as provided by the Global Solar Atlas and Global Wind Atlas. This process allowed to combine the superior spatial resolution of the Global Solar Atlas and Global Wind Atlas (km-scale) with the superior temporal resolution of ERA5 (hourly).

For GHI, we applied a simple additive bias-correction. The bias between the annual average Global Solar Atlas and ERA5 GHI (in kWh/m^2^/year) was calculated for each MSR. This bias was subsequently divided by the number of hours in a year with nonzero irradiation, and the resulting value (in kWh/m^2^/day) was added to the hourly time series of GHI extracted from ERA5 (excluding hours with zero irradiation). This bias was generally very small; across all MSRs, the average absolute difference between annual irradiation levels in MSR centres from the Global Solar Atlas and ERA5 was 3%.

For wind speeds, we used the rank mapping technique (equivalent to empirical quantile mapping^27^, using ranks out of 8760 values as quantiles) to map all hourly wind speed values extracted from ERA5 to a separate target dataset for each MSR. (We note here that the Global Wind Atlas dataset is based on downscaled ERA5 data. The downscaling process used for the Global Wind Atlas allows to resolve local topography-induced corridors of high wind speeds, such as hill ridges, whereas ERA5 grid cells are generally too large to resolve these.) Such a target dataset should have the same average as the Global Wind Atlas average value for the MSR, but also reflect the Weibull-shape distribution of wind speed time series, which is why an additive bias-correction (as for GHI) is not appropriate here (since it does not preserve the Weibull shape). The mappings and target datasets were obtained separately for each country, based on all ERA5 cells linked to MSRs within that country, as follows: If a country has *N* MSRs for wind power, it accordingly has *N* time series of 8760 hourly wind speed values obtained from ERA5. The 8760 values in each time series were assigned a rank between 1 and 8760 (reflecting lowest to highest values within that series). This resulted in *N* wind speed values assigned rank 1, *N* wind speed values assigned rank 2, etc. Subsequently, the *N* values in each rank were correlated against the annual average wind speed from each of the *N* time series, and 8760 linear fits were thus made (one fit through *N* data points for each rank). The bias-correction mapping was then obtained by evaluating those linear fits for each rank at the average wind speeds from the Global Wind Atlas that represents the bias-correction target for each MSR. Thus, *N* target datasets (each containing 8760 values) were obtained from *N* mappings (i.e. the evaluations of 8760 linear fit equations, unique for each country, at *N* target mean wind speeds). Any extracted ERA5 time series could thus be bias-corrected by determining the rank of each value in the time series and subsequently mapping to the target value based on the linear fit for that rank. This process successfully bias-corrects to the target mean while preserving a Weibull shape distribution of wind speeds at each site. This bias-correction was necessary as the annual mean wind speeds computed for each MSR from the Global Wind Atlas were, on average, 45% higher than those obtained from ERA5 due to the latter’s spatial coarseness.

After bias-correction, the GHI and temperature datasets were converted to solar PV capacity factor datasets according to the parameterisations of ref. ^28^, and the 100-m wind speeds were converted to wind turbine capacity factors according to ref. ^15^ (which distinguishes between Class-III, Class-II and Class-I wind turbines based on the average wind speed: less than 7.5 m/s, between 7.5 and 8.5 m/s, or higher than 8.5 m/s, respectively).

The hourly capacity factor profiles (8760 values) for each MSR are provided as metadata of the MSR dataset (see Data Availability). A full list of metadata is provided in the Supplementary Material A.

#### Clustering method

Seeking a balance between computational load and model detail, we propose a clustering approach to group MSRs based on their similarity to one another in addition to the MSR algorithm discussed above.

In this approach, MSRs are grouped based on their CF timeseries. This is executed by using the Euclidean distance between these timeseries to perform *k*-means clustering. The coarseness of the clusters can be controlled by setting the maximum number of clusters according to a modeller’s preferences. Representative parameters for each cluster are determined by taking the sum or, where appropriate, weighted average (by maximum capacity deployable) of those MSRs that make up the cluster. The resulting hourly capacity factor profile is made up by the weighted average production of the individual MSRs at each hour.

This algorithm does not intrinsically consider geographical distance between MSRs as a criterion. However, given the typical spatial correlation observed for VRE potential across certain distances^7^, the clusters typically represent areas that are geographically contiguous or near-contiguous (although constrained by the presence of country borders and exclusion zones). This is shown in Supplementary Material E.

## Results

### Solar PV and wind MSRs across Africa

**Fig. 3:**
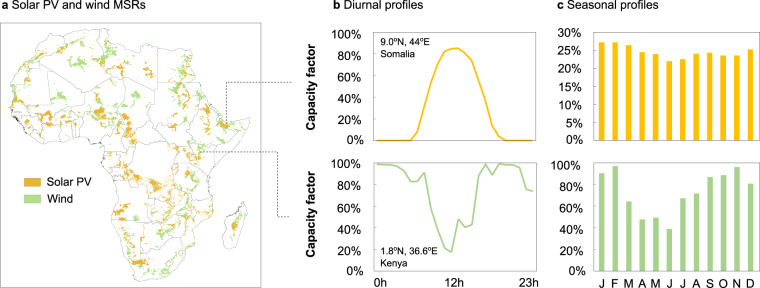
Spatial distribution of solar PV and wind MSRs across Africa. (**a**) A map of the African continent showing all solar PV and wind MSRs screened by LCOE up to a maximum coverage of 5% of a country’s area. (**b**,**c**) Example temporal profiles (diurnal and seasonal) for the two example locations indicated in (**a**). The diurnal example in (**b**) covers the 12th day of March.

The resulting (screened) set of lowest-cost MSRs by country is provided in Fig. 3a (see Data Availability for individual maps on country-level). This screened dataset contains 10,905 MSRs for solar PV across Africa (with an estimated total deployment potential of 4.9 TW at 21.4% average CF) and 7,177 for wind power (3.4 TW at 54.9% average CF). Additionally, we show two examples of local hourly and seasonal profiles (from a solar PV MSR in Somalia and a wind MSR in Kenya) in Fig. 3b,c. Hourly profiles are provided for an example meteorological year, in this case 2018; however, since these are derived from reanalysis datasets (see Methods section “Additional methodological details”), the same analysis can be readily redone with reanalysis data for any other year.

Given countries’ vast disparity in spatial resource distribution, the identified set of MSRs allows for a deeper analysis on the combined effect of resource strength and grid distance on site attractiveness. These two aspects are paramount as the main determinants of the expected LCOE in each MSR are, first, resource strength (the stronger the resource, the higher the yield per unit of capacity, and the lower the LCOE), and second, the distance from existing grid and road infrastructure (the higher this distance, the higher the additional costs to connect power plants to the grid, and the higher the LCOE).

**Fig. 4:**
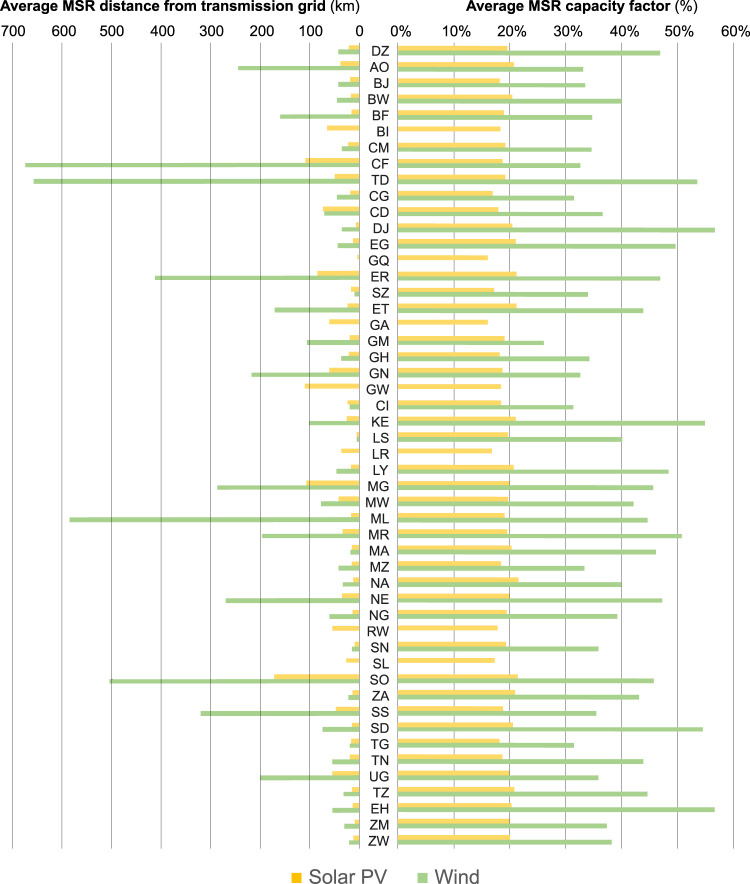
Capacity factor of MSRs as compared to their distance from the transmission grid. The left axis shows the average distance from the transmission grid across all MSRs in a country; the right axis shows the average capacity factor. Averages are weighted by MSR area. Solar PV and wind MSRs differ in (**a**) the spatial divergence of CFs (larger for wind than solar PV) and in (**b**) the distance-from-grid of the cheapest MSRs (higher for wind than solar PV). Country abbreviations denote alpha-2 codes; see Supplementary Table 4 for the list of full names. Countries are ranked vertically according to the alphabetical order of these full names. Note that some countries do not have any viable wind power potential according to the present methodology, hence bars for wind power are omitted for those countries in this graph.

Figure 4 shows the average (MSR area-weighted) distance of each country’s MSRs from the existing transmission grid (left), as well as those MSRs’ expected (area-weighted) average capacity factors (CF, right). This Figure reveals several points of interest. First, the CF of wind power is spatially much more divergent than that of solar PV across countries (a well-known fact, linked to wind power generation scaling with wind speeds to the third power, as opposed to solar PV power generation scaling nearly linearly with irradiation^29^). Second, the distance of MSRs from transmission grid infrastructure is also typically much larger for wind power (close to 160 km) than for solar PV (close to 30 km).

Keeping in mind that the screened MSRs within each country reflect that country’s cheapest options, this signifies that there is generally more economic sense in exploiting remote resources for wind than for solar PV. In other words, paying the “*remoteness premium*” (additional transmission lines and road infrastructure) is more worth the effort for wind than for solar PV, since the extra yield obtained by exploiting excellent far-from-grid wind resources (as opposed to mediocre close-to-grid wind resources) apparently makes up for this premium. Clearly, the same does not apply to solar PV. This appears logical considering the lower spatial resource diversity of solar PV as compared to wind power, which makes far-from-grid investments less attractive.

A follow-up investigation can be performed to assess how substantial the effect of this “remoteness premium” is in cross-country comparisons. For instance, Chad (TD) has among Africa’s best wind power MSRs with an average CF of 54% (Fig. 4, right), but these tend to be very far from existing grid infrastructure (left). How do the costs of these MSRs, which are Chad’s cheapest, compare to the costs of wind MSRs in e.g. Cameroon (CM), where weaker but still viable (at 35% CF) wind resources are found much closer to existing grid infrastructure (by a factor of nearly 20 in terms of distance)?

### Investigating the trade-off between grid proximity and resource strength

**Fig. 5:**
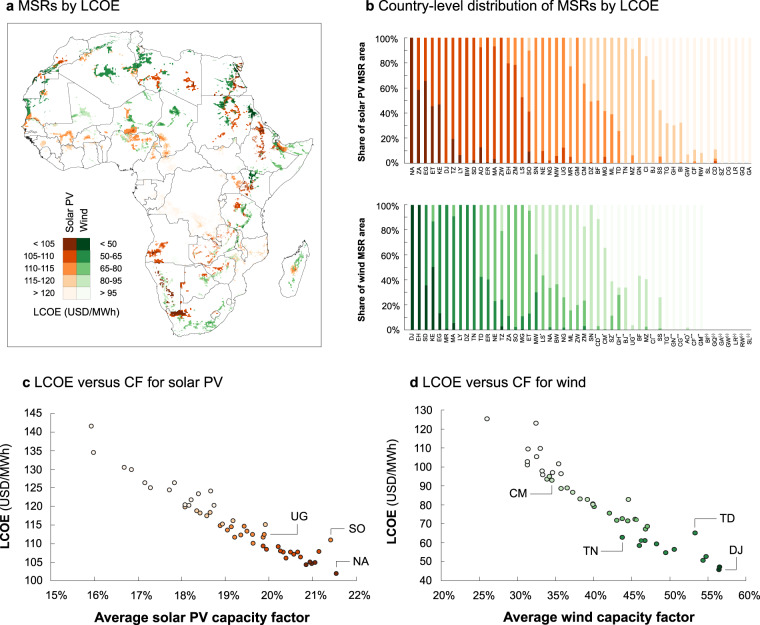
MSRs classified by expected LCOE, including installation costs, operation and maintenance costs, transmission grid extension costs and road network extension costs. (**a**) All-Africa MSRs, screened by LCOE up to coverage of 5% of a country’s area, classified by five LCOE categories from cheaper to costlier. (**b**) The country-level area-weighted distribution of MSRs across these five categories. Country abbreviations denote alpha-2 codes; see Supplementary Table 4 for a list of full names. Countries with comparatively low overall VRE potential are marked with symbols (*), (**) or (***) if total MSR area covered less than 3%, 1% and 0.1% of the country, respectively, and with (−) in case of absence of MSRs in that country. (**c**,**d**) Each country’s average LCOE as function of average CF (averages weighted by MSR area), for solar PV (**c**) and wind (**d**). SO = Somalia, NA = Namibia, UG = Uganda, DJ = Djibouti, TD = Chad, TN = Tunisia, CM = Cameroon.

We visualise all MSRs for solar PV and wind power as classified by their relative cost in Fig. 5a (cost expressed in USD2019). All MSRs were binned into five categories by their expected LCOE (separately for solar PV and wind). Across the African continent, based on the most recent global-level VRE, transmission and road infrastructure costing data for the present-day available to the authors^2^, the LCOEs for MSRs range from 97.7–148.6 USD/MWh for solar PV and from 34.5–127.4 USD/MWh for wind power. Clearly, the lowest-cost MSRs for both VRE types are found in a “boomerang” shape which stretches from West to East across the Sahara and parts of the Sahel, then across the length of the East African Rift from Northeast to Southwest Africa. On the other hand, southern West Africa and large parts of Central Africa are clearly less well-resourced in the African context—although their solar PV potential is still markedly above that of e.g. many European countries.

In Fig. 5b, we show the division of MSRs within each country (by area) across the different LCOE bins, with countries ordered from left to right by average MSR LCOE. Interestingly, a few countries (e.g. Kenya, Djibouti) appear at the high end (i.e. most favourable LCOE in continental comparison) for both resources, whereas others (e.g. Gabon, Equatorial Guinea) find themselves at the low end for both. This suggests that, in the future (in which countries’ power systems are expected to become more interconnected), some specific countries may potentially emerge as typical “VRE hubs” (similarly to how some countries, like Ethiopia and Guinea, are already known as hydropower hubs^5,8^).

The relationship between LCOE and resource strength, and the influence of grid and road infrastructure costs, is elucidated in Fig. 5c,d. Here, we show the (area-weighted) average LCOE versus (area-weighted) average CF by country. (Charts showing the breakdown of this trend by individual MSR are given in Supplementary Material C.) In both cases, a very clear trend of decreasing LCOE with increasing CF is evident, as would be expected. However, in both cases, there are also clear instances of the same CF resulting in diverging LCOEs. This is explained by the difference between near-grid and far-from-grid resources in countries with similar average resource strength.

As an example, the highest-yield MSRs overall for solar PV are found in Namibia and Somalia (Fig. 5c), but Namibia achieves a substantially lower average LCOE: Namibia has more adequate existing grid infrastructure and the “remoteness premium” is correspondingly lower than in Somalia. The Somalian LCOE is, in fact, similar to that expected for solar PV farms in Uganda, which would have a CF of more than a percentage point less, but where existing grid infrastructure is more adequate.

A similar effect can be seen for wind power when looking at the case of Chad (Fig. 5d). Expected capacity factors in Chad would be very close to those of Djibouti, the country achieving the lowest average wind LCOE. Yet, the additional costs for grid connection of Chadian wind farms would be monetarily equivalent to exploiting resources with a CF of roughly 10 percentage points lower but in imminent vicinity of existing grid infrastructure (e.g. as typical for MSRs in Tunisia). However, the costs of these Chadian wind MSRs would still trump those of Cameroon evoked earlier (nearly 30% cheaper in Chad compared to Cameroon).

Generally, it can be concluded from Figs. 3 to 5 that the relative attractiveness of sites for deployment of VRE plants across Africa is determined by a number of factors. The most important factor, generally, is resource strength. For instance, the lowest-cost sites for solar PV and wind are to be found in the countries with the best resource availability (e.g. Namibia, South Africa and Egypt for solar PV, and Djibouti, Sudan and Kenya for wind), cf. Fig. 5b.

When intercomparing countries by site attractiveness, resource strength is therefore generally a good indicator, with the notable exception of countries with very poorly built-out grid infrastructure. For instance, Somalia (for solar PV) and Chad (for wind) have highly attractive sites from a resource point of view, but these sites would be equivalent to mediocre near-grid sites in LCOE terms given the additional costs for grid and road expansion that would be involved), cf. Fig. 5c,d.

When intercomparing sites within a single country, resource strength is also seen to be the most important indicator for site attractiveness for wind power, but importantly, not for solar PV (cf. Figure 4). For solar PV, given its relatively low spatial disparity within each individual country, the best sites (in LCOE terms) simply tend to cluster close to existing grid infrastructure.

## Discussion

In this study, we provide an all-Africa dataset of locations for solar PV and wind park deployment, and their metadata, to serve the energy modelling community. The dataset contains enough information to include the locations in cost-optimisation models for capacity expansion and distinguishes between resources with different *quality* and resources with different *accessibility*.

It is seen that the most attractive locations (in LCOE terms) for solar PV plants tend to cluster near existing grid infrastructure, whereas the most attractive locations for wind power plants are spatially much more widely distributed. The dataset has also provided some insights on the possible compromises between resource quality and grid proximity that may have to be considered for African power systems planning.

Several improvements to the dataset are under consideration for the future. First, as on-the-ground deployment of VRE plants accelerates across Africa, the reanalysis-inferred power generation profiles of the MSRs should be compared to and validated against observed data from actual power plants to validate the robustness of the employed methods. Second, as power grids and road networks are presently in full expansion across Africa to enhance electricity access, the dataset will have to be regularly updated to account for these new realities, which may make locations that are currently relatively inaccessible for grid connection more attractive in the future. Third, as the upfront investment and operating & maintenance costs of solar PV and wind power continue to drop, the relative importance of the “distance-from-grid” criterion vis-à-vis the “resource strength” criterion will shift; typically, the lower the upfront and running costs of VRE, the closer the cheapest MSRs will cluster around available grid infrastructure, even if this means slightly lower capacity factors (see Supplementary Material D). Fourth, the methodology could be extended to offshore wind power, solar CSP, and other types of VRE, e.g. tidal and wave power. Fifth, the methodology could be improved by including transmission grid congestion (i.e. scoring the availability of existing grid infrastructure in terms of available capacity, not by the mere presence of the transmission lines) as a parameter in the creation of MSRs. The current algorithm only scores for proximity to existing grid. As generation capacity gets added to MSRs in concentrated areas where there is an existing grid, the ability for the existing grid to evacuate more power will diminish, requiring additional investment in grid capacity. This should increase the LCOE of the subsequent MSRs, which although are in close proximity to the grid would have to carry additional investment costs. Sixth, we note that the presented dataset used the meteorological year 2018 as basis to calculate hourly power supply time series. For applications in which the correlation between VRE supply and electricity demand *for all individual hours of the year* is of prime importance (e.g. models that run at full 8760-hour temporal resolution), we therefore urge users to consider whether the choice of the year 2018 is adequate or whether another year, or a combination of years into e.g. a “typical meteorological year”, would be better suited. And seventh, the dataset could eventually be extended to cover all other continents as well, allowing for better data validation and more extended statistical analysis of MSR characteristics in space and time.

## Supplementary information

Supplementary Material

## Data Availability

The screened MSRs are available in a public repository on 10.5281/zenodo.7014609 in various formats: (1) country-level georeferenced maps, showing how the screened MSRs align with load centres, roads and transmission infrastructure within a country’s borders, and how their CFs and estimated LCOEs differ across a country’s territory; (2) Excel files (along with their metadata, including hourly profiles) for screened and pre-screened datasets; and (3) GIS shapefiles for screened datasets^30^.

## Code availability

The Python code used to generate the MSRs along with all their metadata, including hourly profiles, as well as the code to perform screening and clustering, is openly available on https://github.com/bhussain89/Model-Supply-Regions-MSR-Toolset.
