# Supplementary material for: An all-Africa dataset of energy model “supply regions” for solar photovoltaic and wind power
Source: Sci Data. 2022 Oct 31;9:664. doi: 10.1038/s41597-022-01786-5 (PMC9622823; doi:10.1038/s41597-022-01786-5)
Supplement: Supplementary file 1 — Supplementary Material [file 41597_2022_1786_MOESM1_ESM.pdf]

**An all-Africa dataset of energy model “supply regions” for solar photovoltaic and wind power: Supplementary Material**

Sebastian Sterl[1,2,3], Bilal Hussain[1], Asami Miketa[1], Yunshu Li[1], Bruno Merven[1,4], Mohammed Bassam Ben Ticha[1,5], Mohamed A. Eltahir Elabbas[1,6], Wim Thiery[2], Daniel Russo[1]

[1] International Renewable Energy Agency (IRENA), Bonn, Germany
[2] Faculty of Engineering, BClimate group, Department HYDR, Vrije Universiteit Brussel, Brussels, Belgium
[3] World Resources Institute (WRI), Regional Hub for Africa, Addis Ababa, Ethiopia
[4] Energy Systems Research Group, University of Cape Town, Cape Town, South Africa
[5] International Atomic Energy Agency (IAEA), Vienna, Austria
[6] Institute for Research in Technology (IIT), ICAI School of Engineering, Comillas Pontifical University, Madrid, Spain

# Contents

*A. Full list of metadata provided with MSRs* .......... 2
*B. Costs used for LCOE calculations* .......... 4
*C. LCOE versus capacity factor: full plots* .......... 5
*D. Effect of future CAPEX and OPEX reductions* .......... 7
*E. Clustering approach* .......... 8

# A. Full list of metadata provided with MSRs

A full list of the metadata provided for all MSRs in the data files is shown in Table 1 for solar PV and in Table 2 for wind power.

*Table 1: Metadata provided for all solar PV MSRs*

| Parameter | Explanation | Unit |
|---|---|---|
| MSR_ID | ID assigned to each MSR | |
| Longitude | Longitude of MSR center | ºE |
| Latitude | Latitude of MSR center | ºN |
| AreakM2 | MSR area | $m^2$ |
| CapacityMW | Maximum deployable capacity in MSR | MW |
| RoadDist | Distance from road infrastructure | km |
| TD_Dist_gf | Distance from nearest transmission line | km |
| T_Dist_gf | Distance from nearest distribution line | km |
| D_Dist_gf | Distance from nearest transmission/distribution line | km |
| SubstnDist | Distance from nearest substation (from OpenStreetMap [1]) | km |
| Load_dst | Distance from nearest urban area (based on World Urban Areas dataset [2]) | km |
| City_name | Name of nearest urban area | |
| City_Pop | Population of nearest urban area | |
| CtLst100kM | Count of urban areas lying within 100 km radius of MSR center | |
| CtCnt100kM | Comma-separated name list of urban areas lying within 100 kms radius of MSR center. If >10, it displays “Above 10 cities”. | |
| PopIn100kM | Sum of population of all urban areas within 100 km | |
| CtryName | Name of country containing MSR | |
| GHIkWhm2d | Mean GHI according to Global Solar Atlas, spatially averaged across MSR | $kWh/m^2/day$ |
| RawERAmean | Mean GHI extracted from ERA5 grid cell closest to MSR center | $kWh/m^2/day$ |
| CorAdderWh | Additive bias-correction factor (added to each nonzero hourly GHI value in the nearest-neighbour ERA5 datapoint) | $kWh/m^2/day$ |
| CF | Annual average capacity factor (including assumed 8% losses, consisting of 4% outage and 4% inverter and cable losses [3]) | |
| Y_GWh | Yearly yield from maximum deployable capacity | GWh |
| sLCOE-MWh | LCOE of power plant assets | USD/MWh |
| tLCOE-MWh | LCOE of transmission/grid assets | USD/MWh |
| tCAPEX-kW | Transmission/grid asset CAPEX | USD/kW |
| rLCOE-MWh | LCOE of road assets | USD/MWh |
| rCAPEX-kW | Road asset CAPEX | USD/kW |
| LCOE-MWh | Full LCOE | USD/MWh |
| trCAPEX-kW | Transmission/grid + road asset CAPEX | USD/kW |
| H1...H8760 | Hourly capacity factor (%) for one full year (not counting the losses reported under “CF”) | |

*Table 2: Metadata provided for all wind power MSRs*

| **Parameter** | **Explanation** | **Unit** |
|---|---|---|
| MSR_ID | ID assigned to each MSR | |
| Longitude | Longitude of MSR center | ºE |
| Latitude | Latitude of MSR center | ºN |
| AreakM2 | MSR area | $m^2$ |
| CapacityMW | Maximum deployable capacity in MSR | MW |
| RoadDist | Distance from road infrastructure | km |
| TD_Dist_gf | Distance from nearest transmission line | km |
| T_Dist_gf | Distance from nearest distribution line | km |
| D_Dist_gf | Distance from nearest transmission/distribution line | km |
| SubstnDist | Distance from nearest substation (from OpenStreetMap [1]) | km |
| Load_dst | Distance from nearest urban area (based on World Urban Areas dataset [2]) | |
| City_name | Name of nearest urban area | |
| City_Pop | Population of nearest urban area | |
| CtLst100kM | Count of urban areas lying within 100 km radius of MSR center | |
| CtCnt100kM | Comma-separated name list of urban areas lying within 100 kms radius of MSR center. If >10, it displays "Above 10 cities". | |
| PopIn100kM | Sum of population of all urban areas within 100 km | |
| CtryName | Name of country containing MSR | |
| MeanSpeed | Mean wind speed according to Global Wind Atlas, spatially averaged across MSR | m/s |
| IEC_Class | Wind turbine class | |
| ERA_Wspeed | Mean wind speed (m/s) extracted from ERA5 grid cell closest to MSR center | m/s |
| CF100m | Annual average capacity factor (including assumed 17% losses, consisting of 2% outage and 15% array and wake losses [3]) for 100m-hub height turbine | |
| Y_GWh100m | Yearly yield from maximum deployable capacity (GWh) | GWh |
| sLCOE-MWh | LCOE of power plant assets (USD/MWh) | USD/MWh |
| tLCOE-MWh | LCOE of transmission/grid assets (USD/MWh) | USD/MWh |
| tCAPEX-kW | Transmission/grid asset CAPEX (USD/kW) | USD/kW |
| rLCOE-MWh | LCOE of road assets (USD/MWh) | USD/MWh |
| rCAPEX-kW | Road asset CAPEX (USD/kW) | USD/kW |
| LCOE-MWh | Full LCOE (USD/MWh) | USD/MWh |
| trCAPEX-kW | Transmission/grid + road asset CAPEX (USD/kW) | USD/kW |
| H1...H8760 | Hourly capacity factor (%) for one full year (not counting the losses reported under "CF100m") | |

## B. Costs used for LCOE calculations

The levelized cost of electricity (LCOE) of solar PV and wind power plants deployed in the identified MSRs is composed of three separate terms:

$$LCOE = LCOE_s + LCOE_t + LCOE_r,$$

Where $LCOE_s$ refers to the investment and operation & maintenance (O&M) costs of the power plant itself; $LCOE_t$ refers to the costs for transmission infrastructure, and $LCOE_r$ refers to the costs for road infrastructure. Each separate LCOE term is calculated as follows:

$$LCOE = \frac{\sum_y \frac{(I_y + M_y)}{(1+r)^y}}{\sum_y \frac{E_y}{(1+r)^y}},$$

where $y$ represents the year of the asset's lifetime ($0 \leq y \leq Y$, with $Y$ the plant's lifetime), $I_y$ are the initial (overnight) costs related to construction of the asset in each year $y$, $M_y$ are the operational and maintenance costs in each year $y$, $E_y$ is the total electricity generated by the plant in each year $y$, and $r$ is the discount rate.

*Table 3: The parameters used in the calculation of the LCOE of solar PV and wind power plants deployed in the identified MSRs. All costs expressed in USD2019 (note that the cited source may have provided data in a different currency year).*

| Cost type | Value | Unit | Source | Notes |
|---|---|---|---|---|
| Solar PV investment costs | 1070 | USD/kW | [4] | |
| Solar PV O&M costs | 53.5 (fixed)<br>4 (variable) | USD/kW/year<br>USD/MWh | [3] | |
| Solar PV lifetime | 25 | years | [3] | |
| Wind power investment costs | 1338 (Class-I)<br>1552 (Class-II)<br>1819 (Class-III) | USD/kW | [3] | |
| Wind power O&M costs | 64.2 | USD/kW/year | [3] | |
| Wind turbine lifetime | 25 | years | [3] | |
| Transmission line investment costs | 1059.3 | USD/MW evacuated/km | [3] | MW evacuated based on the maximum deployable capacity in each MSR |
| Transmission line O&M costs | 0 | USD/km/year | [3] | |
| Transmission line lifetime | 40 | years | [3] | |
| Road extension costs | 435490 | USD/km | [3] | |
| Road O&M costs | 0 | USD/km/year | [3] | |
| Road lifetime | 25 | years | [3] | |
| Substation costs | 75970 | USD/substation | [3] | One substation to be deployed at each plant for low- to high-voltage transformation; one substation to be deployed at transmission line end |
| Discount rate | 10% | - | [5] | |

## C. LCOE versus capacity factor: full plots

Figure 1 and Figure 2 display the full scatterplots of MSR LCOE versus average capacity factor. Each point represents an MSR; point sizes are proportional to MSR sizes (area, or equivalently the maximum deployable capacity in MW in each MSR). Colours represent the “power pool” that the country in question belongs to; this classification is given in Table 4.

.

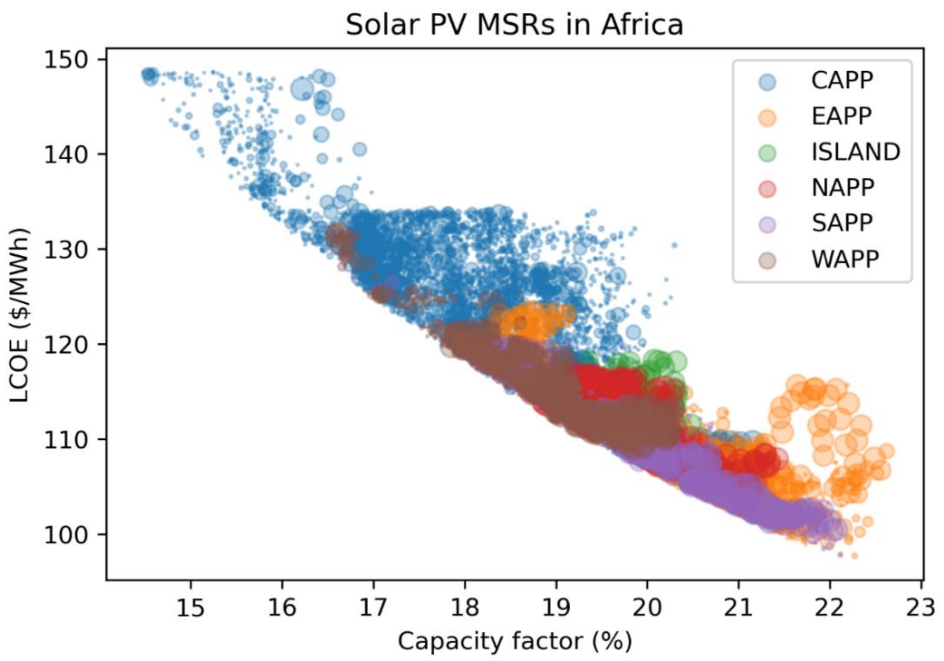


*Figure 1: Full set of solar PV MSR LCOE values versus average capacity factor. Point sizes are proportional to MSR area.*

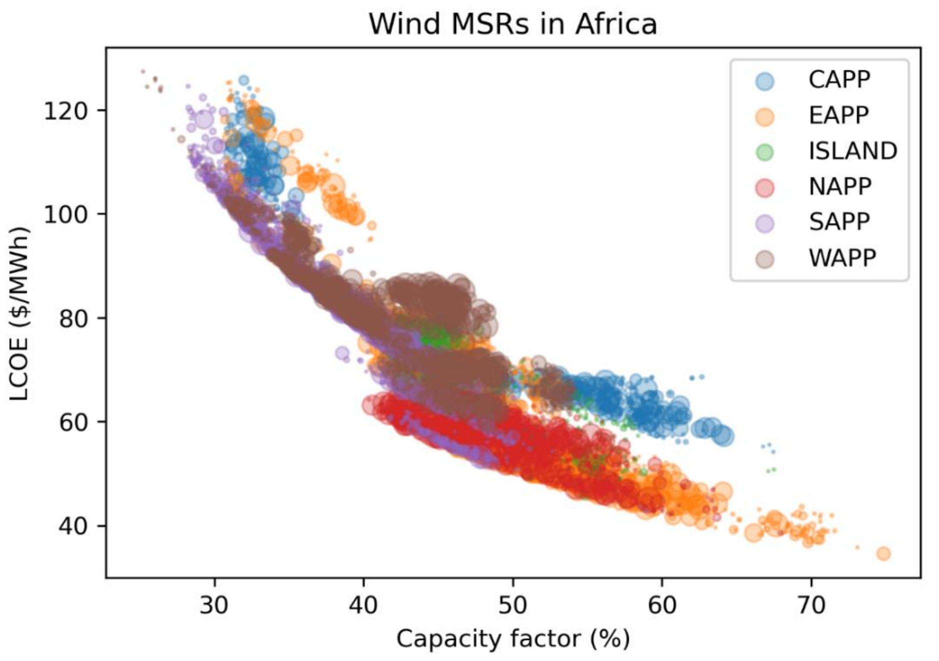


*Figure 2: Full set of wind MSR LCOE values versus average capacity factor. Point sizes are proportional to MSR area.*

*Table 4: Countries/regions considered in this study, including their alpha-2 country code and the Power Pool to which they were assigned (by the authors; note that these designations are meant to represent geographical regions, and do not correspond 100% to the legal entities known as "power pools", as e.g. some countries are legally not a member of any, and others are members of multiple). NAPP = North African Power Pool, CAPP = Central African Power Pool, EAPP = Eastern Africa Power Pool, SAPP = Southern African Power Pool, WAPP = West African Power Pool.*

| **Country/region** | **Alpha-2 code** | **Power Pool** |
|---|---|---|
| Algeria | DZ | NAPP |
| Angola | AO | CAPP |
| Benin | BJ | WAPP |
| Botswana | BW | SAPP |
| Burkina Faso | BF | WAPP |
| Burundi | BI | CAPP |
| Cameroon | CM | CAPP |
| Central African Republic | CF | CAPP |
| Chad | TD | CAPP |
| Congo | CG | CAPP |
| Democratic Republic of the Congo | CD | CAPP |
| Djibouti | DJ | EAPP |
| Egypt | EG | EAPP |
| Equatorial Guinea | GQ | CAPP |
| Eritrea | ER | EAPP |
| Eswatini | SZ | SAPP |
| Ethiopia | ET | EAPP |
| Gabon | GA | CAPP |
| Gambia | GM | WAPP |
| Ghana | GH | WAPP |
| Guinea | GN | WAPP |
| Guinea-Bissau | GW | WAPP |
| Côte d'Ivoire | CI | WAPP |
| Kenya | KE | EAPP |
| Lesotho | LS | SAPP |
| Liberia | LR | WAPP |
| Libya | LY | NAPP |
| Madagascar | MG | n/a (ISLAND) |
| Malawi | MW | SAPP |
| Mali | ML | WAPP |
| Mauritania | MR | NAPP |
| Morocco (see note on EH) | MA | NAPP |
| Mozambique | MZ | SAPP |
| Namibia | NA | SAPP |
| Niger | NE | WAPP |
| Nigeria | NG | WAPP |
| Rwanda | RW | CAPP |
| Senegal | SN | WAPP |
| Sierra Leone | SL | WAPP |
| Somalia | SO | EAPP |
| South Africa | ZA | SAPP |
| South Sudan | SS | EAPP |

| | | |
|---|---|---|
| Sudan | SD | EAPP |
| Togo | TG | WAPP |
| Tunisia | TN | NAPP |
| Uganda | UG | EAPP |
| United Republic of Tanzania | TZ | EAPP |
| Western Sahara (analysed as separate region for the purposes of this study) | EH | NAPP |
| Zambia | ZM | SAPP |
| Zimbabwe | ZW | SAPP |

## *D. Effect of future CAPEX and OPEX reductions*

Given the substantial reductions in capital and operational expenses (CAPEX and OPEX) for solar PV and wind power expected over the coming years, the results of this study—in particular, the compromise between exploiting good resources and paying the "remoteness premium" when attempting to screen the lowest-cost sites—may shift in the future. Assuming no changes in infrastructure costs for transmission lines, substations, and road construction, a reduction in CAPEX and OPEX of VRE would theoretically tend to shift the most favourable MSRs (in LCOE terms) somewhat closer to grid infrastructures, with the avoided remoteness premium making up for losses in average yield. The question is whether this effect is substantial, marginal, or non-existent.

A sensitivity test was run on the basis of predicted CAPEX and OPEX values for 2040 for solar PV and wind. Based on historical learning rates observed for these sources [4], for this test, CAPEX were assumed to drop by 50% between the present-day and 2040 for both solar PV and wind, and OPEX were assumed to drop by 50% for solar PV and by 60% for wind. The results are summarised in Table 5, which shows the average gains in grid closeness of MSRs (when passing from present-day cost assumptions to 2040 cost assumptions), alongside the corresponding average compromises in CFs. This is done both for the African average, as well as for the three countries with the strongest geographical shift in MSRs when passing from present-day to 2040 costs.

*Table 5: Changes in geographical location of MSRs (expressed in average reduction in distance from the grid) and yield of these MSRs (expressed in compromise in average CF) when changing from present-day to 2040 assumptions on the CAPEX and OPEX of solar PV and wind power.*

| | | MSR shift towards grid infrastructure | Compromise in average MSR capacity factor |
|---|---|---|---|
| **Solar PV** | **Africa-wide** | **0.8 km** | **-0.1 pp** |
| | Malawi (strongest shift) | 20.9 km | -0.3 pp |
| | Gabon (2nd strongest) | 20.6 km | -0.4 pp |
| | DRC (3rd strongest) | 13.1 km | -0.2 pp |
| **Wind** | **Africa-wide** | **50.2 km** | **-0.9 pp** |
| | Mali (strongest shift) | 299.4 km | -5.1 pp |
| | Chad (2nd strongest) | 179.2 km | -3.9 pp |
| | Niger (3rd strongest) | 137.3 km | -1.9 pp |

It can be concluded that the effect, on average, is relatively small for solar PV, whose MSRs already tend to cluster around grid infrastructure even under present-day costs, an effect which would be only slightly strengthened by further cost drops. The effect is more important for wind, although strongly diverging on a country-by-country basis, with some countries

seeing nearly no geographical shift of MSRs and others seeing substantial changes. A few clear outliers can be identified for wind power: these are countries straddling the Sahelian belt, which has excellent wind resources but mostly at several hundreds of kilometres from existing grid infrastructure.

## E. Clustering approach

The clustering approach developed to complement the MSR identification algorithm is described in Methods. In Figure 3, we show an example of the results of the clustering in a geographical sense. We use the example of Mali, which had 145 separate MSRs for solar PV and 131 separate MSRs for wind (based on the screening criterion of maximum 5% coverage of a country area mentioned in the main text). Here, the MSRs were grouped into five clusters for both solar PV and wind, each with their own maximum deployable capacity, weighted average cost parameters and weighted average capacity factor time series.

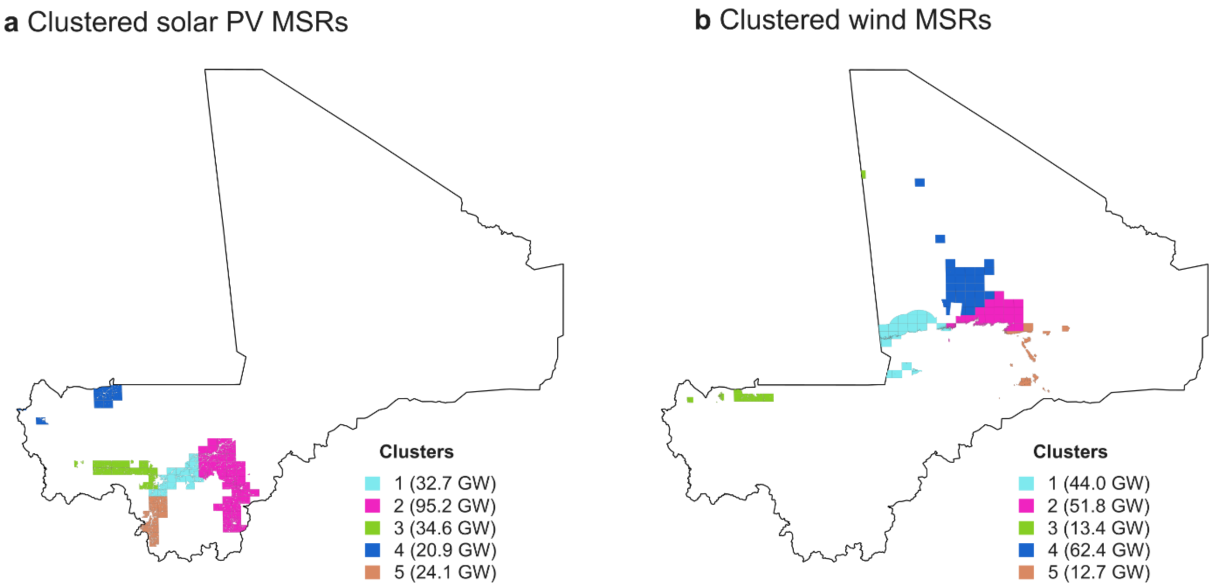


*Figure 3: A geographical view of the clusters developed for Mali for solar PV (a) and wind power (b). MSRs were clustered into five clusters in both cases. Numbers in brackets indicate the maximum deployable capacity across each cluster (GW).*

### Bibliography


1. OpenStreetMap®. OpenStreetMap® from ©OpenStreetMap Contributers. (2018).

2. Patterson, T. & Kelso, N. V. World Urban Areas, LandScan, 1:10 million (2012). [Shapefile]. North American Cartographic Information Society. https://maps.princeton.edu/catalog/stanford-xg070wh7159.

3. IRENA and LBNL. *Renewable Energy Zones for the Africa Clean Energy Corridor - Multi-Criteria Analysis for Planning Renewable Energy*. https://www.irena.org/-

/media/Files/IRENA/Agency/Publication/2015/IRENA-LBNL_Africa-RE-_CEC_2015.pdf (2015).

4. IRENA. *Renewable Power Generation Costs in 2020*. https://www.irena.org/publications/2021/Jun/Renewable-Power-Costs-in-2020 (2021).
5. IRENA. *Planning and prospects for renewable power: Eastern and Southern Africa*. https://www.irena.org/publications/2021/Apr/Planning-and-prospects-for-renewable-power-Eastern-and-Southern-Africa (2021).
